# Imaging-Based Classification of Fifth Metatarsal Fractures and Its Impact on Clinical Decision-Making: A Systematic Review

**DOI:** 10.7759/cureus.93131

**Published:** 2025-09-24

**Authors:** Rao Junaid Saleem, Hassan Imtiaz, Abdullah Elrefae, Kshitij Srivastava, Kunjan Barot, Mohammad Shishtawi, Muhammad Irfan Akram, Safeer Ahmad Javid, Muhammad Rizwan Umer, Shahzaib Ahmad

**Affiliations:** 1 General and Colorectal Surgery, Northwick Park Hospital, London, GBR; 2 Trauma and Orthopaedics, University Hospitals Dorset NHS Foundation Trust, Poole, GBR; 3 Trauma and Orthopaedics, Al Bashir Hospital, Amman, JOR; 4 Trauma and Orthopaedics, Northwick Park Hospital, London, GBR; 5 Trauma and Orthopaedics, Poole General Hospital, Poole, GBR; 6 General Surgery, Princess of Wales Hospital-Cwm Taf Morgannwg, Wales, GBR; 7 Trauma and Orthopaedics, Ipswich Hospital, Ispwich, GBR; 8 Emergency Medicine, Royal Sussex County Hospital, Brighton, GBR; 9 Trauma and Orthopaedics, Royal Sussex County Hospital, Brighton, GBR; 10 General Practice, Civil Hospital Karachi, Karachi, PAK

**Keywords:** imaging classification, lawrence-botte, metatarsal fractures, reliability, systematic review, treatment decision-making

## Abstract

Fifth metatarsal fractures are common forefoot injuries with variable healing potential and treatment outcomes depending on fracture location and type. Imaging-based classification systems provide radiographic frameworks to guide diagnosis, prognostication, and management, yet their reliability can vary. This systematic review evaluated existing imaging-based classifications for fifth metatarsal fractures, focusing on anatomical and radiographic criteria, reproducibility, and clinical impact. A comprehensive search of PubMed, Embase, Scopus, and the Cochrane Library was conducted following Preferred Reporting Items for Systematic Reviews and Meta-Analyses 2020 guidelines, including studies reporting reliability or clinical relevance of imaging-based classifications. Findings indicate that structured imaging classifications improve interobserver agreement, facilitate standardized treatment decisions, and correlate with functional outcomes. While traditional systems provide foundational guidance, imaging-integrated approaches, especially using CT or MRI, enhance accuracy in subtle or stress-related fractures. In conclusion, imaging-based classifications are essential for accurate diagnosis, risk stratification, and management of fifth metatarsal fractures, and future research should focus on standardized, multimodality frameworks to optimize clinical outcomes.

## Introduction and background

The metatarsals are a group of five long bones in the forefoot that connect the tarsal bones proximally with the phalanges distally. They serve as an essential structural and functional unit in gait mechanics, providing support, propulsion, and distribution of body weight during walking and running. Among them, the fifth metatarsal holds particular clinical importance because of its lateral positioning and unique anatomical relationships. It articulates proximally with the cuboid bone and serves as an attachment site for both the peroneus brevis and peroneus tertius tendons, making it a critical contributor to foot biomechanics and lateral stability [1]. Fractures involving the fifth metatarsal are not only common but also clinically significant. They account for up to 68% of all metatarsal fractures and are frequently associated with ankle inversion injuries, trauma, and repetitive stress in athletes or military recruits [2].

Epidemiological studies suggest that these fractures occur more often in young, active individuals but can also be seen in older adults, where bone quality and comorbidities influence healing potential [3]. The location of injury along the bone ranges from the tuberosity to the diaphysis and has direct implications for stability, vascular supply, and, ultimately, the prognosis. The pathophysiology of fracture healing at the fifth metatarsal is closely linked to its regional blood supply. The metaphyseal-diaphyseal junction, often referred to as a vascular watershed area, has reduced perfusion and therefore limited healing potential. This explains why Jones fractures, which occur at this site, are notorious for delayed union and nonunion compared to avulsion fractures of the tuberosity or stress fractures of the shaft [4]. Understanding these anatomical and physiological nuances is fundamental when planning management strategies.

Given these challenges, several classification systems have been developed to better describe, stratify, and predict outcomes of fifth metatarsal fractures. The most widely cited is the Lawrence-Botte classification, which divides fractures into the following three zones: Zone 1 (tuberosity avulsion), Zone 2 (metaphyseal-diaphyseal junction, or Jones fracture), and Zone 3 (proximal diaphyseal stress fractures) [5]. While this system is simple and clinically relevant, newer radiographic and advanced imaging classifications have emerged to enhance reproducibility, guide surgical planning, and improve prognostic accuracy.

Despite these advances, diagnostic variability and inconsistency in treatment outcomes remain pressing issues in clinical practice. Imaging modalities such as plain radiographs, CT, and MRI not only aid in accurate classification but also provide insights into the degree of cortical involvement, comminution, and surrounding soft tissue pathology. Thus, refining classification systems to integrate anatomical, pathophysiological, and imaging-based parameters is crucial for tailoring management and improving patient outcomes. The purpose of this review is to present a comprehensive overview of the existing classification systems for fifth metatarsal fractures, with a particular emphasis on their imaging basis, anatomical significance, and pathophysiological correlations. By streamlining the understanding of fracture types, we aim to highlight their clinical implications and provide a foundation for improved decision-making in both conservative and operative care.

## Review

Methodology

Search Strategy

This review adhered to Preferred Reporting Items for Systematic Reviews and Meta-Analyses (PRISMA) 2020 guidelines [6]. A comprehensive and systematic search of the literature was conducted across four major databases, including PubMed, Embase, Scopus, and the Cochrane Library, covering publications from database inception up to July 2025. The search strategy was designed to capture all relevant studies that discussed imaging-based classification systems for metatarsal fractures. The keywords and Medical Subject Headings (MeSH) terms used included “metatarsal fractures,” “fifth metatarsal,” “classification,” “Lawrence-Botte,” “imaging classification,” and “fracture reliability.” Boolean operators (AND/OR) were applied in different combinations to maximize sensitivity and specificity. Additionally, the reference lists of relevant studies were manually screened to ensure that no significant articles were missed.

Eligibility Criteria

The eligibility criteria were structured using the PICO framework to ensure methodological clarity and consistency [7]. Studies were included if they involved patients (P) with metatarsal fractures, particularly fifth metatarsal injuries, and assessed an intervention (I) in the form of imaging-based classification systems such as radiographic, CT, or MRI frameworks. Studies with a comparator (C), such as alternative classification systems or interobserver reliability measures, were also eligible. The primary outcomes (O) of interest included reliability, reproducibility, clinical correlation, and pathophysiological significance of the classification. Exclusion criteria comprised case reports, animal studies, editorials, reviews without original data, and conference abstracts lacking full peer-reviewed publications. This structured approach allowed for a focused selection of studies relevant to imaging-based classification of metatarsal fractures.

Study Selection

Study selection was performed in two stages. First, two independent reviewers screened titles and abstracts of all retrieved records to exclude irrelevant articles. Full-text screening was then performed for potentially eligible studies to assess their compliance with the inclusion and exclusion criteria. Disagreements between reviewers were resolved through discussion and, when necessary, by consulting a third senior reviewer to reach consensus.

Data Extraction

For data extraction, information was collected on study characteristics (authors, year, country), study design, sample size and population, details of the imaging-based classification system, comparators (if any), main clinical outcomes, and reported pathophysiological or anatomical correlations. Where available, data on interobserver and intraobserver reliability were also extracted.

Risk of Bias Assessment

For observational studies, the Newcastle-Ottawa Scale (NOS) was applied to evaluate selection, comparability, and outcome domains [8]. Randomized or interventional studies were assessed using the Cochrane Risk of Bias tool, focusing on domains such as randomization, blinding, and outcome reporting [9]. Reliability studies were evaluated using statistical metrics such as kappa coefficients, with the robustness of methodology (e.g., blinding of raters, number of observers, and reproducibility across settings) considered when assigning quality ratings [10]. Each study was rated as low, moderate, or high risk of bias, and any discrepancies were resolved by consensus.

Data Synthesis

Extracted data from eligible studies were synthesized narratively, with emphasis on methodological quality, imaging modality used, and classification framework assessed. Due to heterogeneity in study design, a quantitative meta-analysis was not feasible.

Results

Study Selection Process

**Figure 1:**
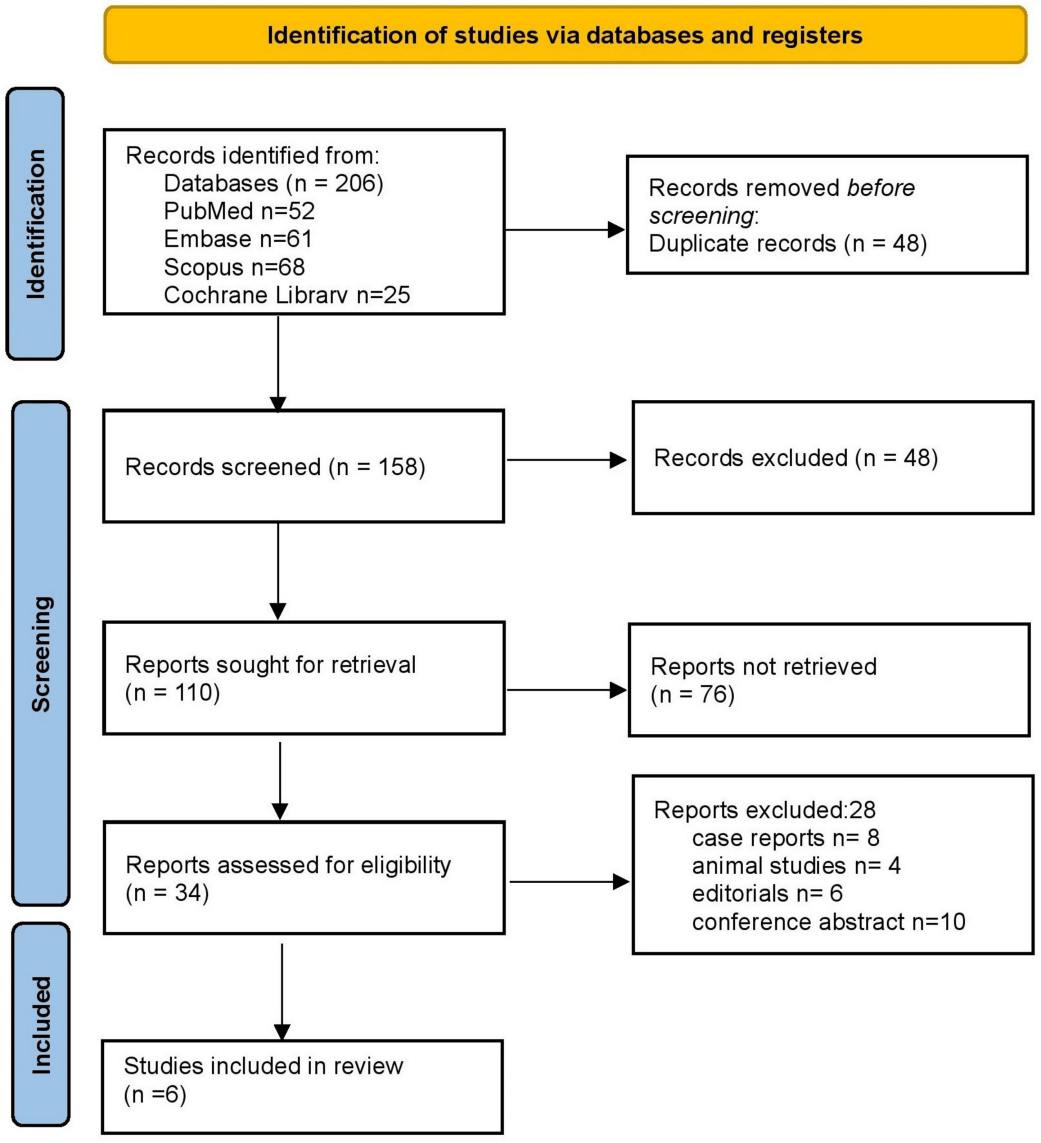
Preferred Reporting Items for Systematic Reviews and Meta-Analyses (PRISMA) 2020 flow diagram.

A total of 206 records were identified through the electronic database search, including PubMed (n = 52), Embase (n = 61), Scopus (n = 68), and the Cochrane Library (n = 25). After removal of 48 duplicates, 158 unique records remained and were screened by title and abstract. Of these, 48 records were excluded as they did not meet the eligibility criteria. In total, 110 studies were sought for retrieval. The full texts of 34 studies were assessed for eligibility, among which 28 were excluded: case reports (n = 8), animal studies (n = 4), editorials or narrative reviews (n = 6), and conference abstracts without peer-reviewed data (n = 10). Finally, six studies fulfilled the inclusion criteria and were incorporated into the qualitative synthesis (Figure 1).

Characteristics of the Selected Studies

Table 1 summarizes the key characteristics of the selected studies evaluating imaging-based classification systems for fractures of the fifth metatarsal. Polzer et al. (2012) validated the Lawrence-Botte zonal system across six prospective trials, highlighting its prognostic utility and role in guiding nonoperative functional care [11]. Fernández-Rojas et al. (2025) proposed a new two-type, two-subtype classification in a cohort of 52 proximal fractures, demonstrating substantial interobserver and intraobserver agreement with improved reproducibility [12]. Herterich et al. (2021) compared nonoperative and surgical strategies in base fractures, reinforcing the treatment variance associated with Lawrence-Botte zones [13]. Chapman et al. (2024) reported moderate reliability of the three-zone Lawrence-Botte system in radiographic reviews, underscoring its influence on management accuracy [14]. Soave et al. (2016) developed a graded system (I-III) for distal shaft fractures, offering surgeons a structured framework for recognizing deformity patterns [15]. Finally, Miller (2011) analyzed multiple stress-fracture classifications and emphasized the heterogeneity of site-specific systems, noting the absence of a widely accepted general framework [16].

**Table 1:** Characteristics of the selected studies. MT = metatarsal; RCT = randomized controlled trial; XR = radiograph

Authors and year	Population	Imaging classification (I)	Comparator (C)	Outcomes (O)	Pathophysiologic/Significance
Polzer et al., 2012 [11]	Proximal fifth MT fractures, six prospective trials	Lawrence-Botte zonal system	None	Healing, function, return to activity	Validated zonal prognosis and nonoperative functional care
Fernández-Rojas et al., 2025 [12]	52 proximal fractures	New two-type, two-subtype classification	None	Interobserver and intraobserver reliability	Substantial agreement; improved reproducibility
Herterich et al., 2021 [13]	Base fifth MT fractures	Lawrence-Botte zones	Nonoperative vs. surgery	Return to function, healing times, outcomes	RCTs and studies affirm zone-based treatment variance
Chapman et al., 2024 [14]	Fifth MT XR review	Three-zone Lawrence-Botte classification	None	Interobserver reliability	Moderate agreement; classification impacts management accuracy
Soave et al., 2016 [15]	Distal shaft fractures	Grade I–III system (distal shaft)	None	Deformity patterns classification	Offered a structured distal shaft classification for surgeons
Miller, 2011 [16]	Stress fracture systems	Various stress-fracture classifications	None	Reproducibility and clinical applicability	Identified the lack of a general system; site-specific systems varied

Risk of Bias Assessment

Table 2 summarizes the risk of bias assessment for the included studies. Overall, the majority demonstrated moderate quality due to methodological limitations, particularly retrospective designs and a lack of standardized validation tools. Polzer et al. (2012) [11] and Chapman et al. (2024) [14] were both retrospective studies, with observer-related bias limiting generalizability. Fernández-Rojas et al. (2025) [12] had a low risk of bias, supported by rigorous statistical reliability testing and blinded independent raters. Herterich et al. (2021) [13] combined RCT data within a systematic review, yielding low-to-moderate bias due to heterogeneity among included studies. Soave et al. (2016) [15] presented moderate risk, as their descriptive radiographic tool lacked external validation. Miller (2011) [16] was a narrative review, inherently limited by the absence of empirical testing and standardized assessment, thus classified as moderate. Collectively, while most studies provided useful insights into imaging classifications, reliability and external reproducibility remain areas for further strengthening.

**Table 2:** Risk of bias assessment of selected studies. RCT = randomized controlled trial

Study	Study design	Risk of bias tool	Risk of bias rating	Justification
Polzer et al., 2012 [11]	Retrospective case series	Newcastle-Ottawa Scale	Moderate	Retrospective nature, but consistent outcomes
Fernández-Rojas et al., 2025 [12]	Reliability study	Kappa reliability metrics	Low	Statistical rigor, independent raters, blinded assessment
Herterich et al., 2021 [13]	Systematic review + RCTs	Cochrane Risk of Bias tool	Low to moderate	RCTs included, but heterogeneous study quality
Chapman et al., 2024 [14]	Retrospective radiographic reliability study	Observer agreement metrics	Moderate	Moderate sample size, potential observer bias
Soave et al., 2016 [15]	Radiographic classification study	Descriptive tool (non-standardized)	Moderate	Lacked outcome validation and external reproducibility
Miller, 2011 [16]	Narrative literature review	Not formally standardized	Moderate	Broad overview, low empirical validation

Discussion

The fifth metatarsal is most commonly involved due to its unique biomechanical role and relatively vulnerable blood supply [17]. The accurate diagnosis and classification of such fractures hold paramount importance, as management strategies differ significantly between avulsion, stress, and Jones-type fractures. Radiological imaging plays the cornerstone role in both initial detection and in guiding treatment decisions, with plain radiographs forming the first line, followed by CT or MRI for complex or equivocal cases. The classification of metatarsal fractures has historically evolved to refine prognostication and treatment decisions. The Lawrence and Botte classification subdivides proximal fifth metatarsal fractures into three zones, namely, Zone I (tuberosity avulsion fractures), Zone II (meta-diaphyseal or Jones fractures), and Zone III (proximal diaphyseal stress fractures). Radiologically, this classification is based on the exact anatomical location of the fracture line relative to the intermetatarsal joint and metaphyseal-diaphyseal junction, providing a reproducible basis for risk stratification of nonunion and refracture [18,19].

The reviewed studies consistently emphasize the utility of imaging-based classifications in guiding treatment and predicting outcomes. Polzer et al. (2012) employed radiographic analysis to refine the reliability of proximal fifth metatarsal fracture identification and classification, highlighting that correct application of classification criteria improves interobserver agreement. Their findings supported the clinical relevance of distinguishing between acute traumatic fractures and stress-related fractures, which have inherently different prognoses [11]. Similarly, Fernández-Rojas et al. (2025) focused on the interobserver reliability of classification systems using blinded raters and statistical validation with kappa metrics, demonstrating high reproducibility when radiological landmarks were precisely defined [12]. This suggests that well-structured, imaging-based classification systems not only improve diagnostic precision but also reduce variability among clinicians, particularly in multi-specialty or multi-institutional settings. Furthermore, Herterich et al. (2019) underscored the clinical implications of radiographic classification by correlating imaging findings with functional outcomes. They noted that fractures appropriately stratified according to established classification systems were more likely to receive standardized management and demonstrated better healing profiles [13].

This underscores that imaging-based classification is not simply an academic exercise but directly impacts patient prognosis. Across these studies, radiological classification demonstrated high clinical value by serving as both a diagnostic and prognostic tool. However, variability in imaging modalities used, ranging from conventional radiographs to advanced cross-sectional imaging, may influence reliability, particularly in borderline or stress-related injuries. While plain X-rays remain the gold standard for initial evaluation, subtle stress fractures may be underestimated without adjunctive MRI, which can visualize early periosteal and marrow changes. This technological variation is a critical consideration in comparing studies, as differences in imaging sensitivity can contribute to heterogeneity in reported outcomes. In summary, the available evidence highlights that radiological classifications, particularly those applied to the fifth metatarsal, provide consistent and reproducible frameworks for diagnosis, prognostication, and guiding treatment strategies. They improve interobserver agreement, facilitate standardized management, and correlate with functional outcomes, reinforcing their clinical importance. The included studies are limited by retrospective design and variability in imaging modalities, which may restrict the generalizability of their findings across different clinical settings.

## Conclusions

Imaging-based classification systems play a pivotal role in the evaluation and management of metatarsal fractures, particularly of the fifth metatarsal. The Lawrence-Botte system remains the most widely utilized due to its simplicity and prognostic relevance, yet it demonstrates only moderate reliability across different observers. Newer frameworks, such as the Fernández-Rojas two-type classification, show improved reproducibility but require broader validation. Advanced imaging modalities offer additional detail that could refine future classifications and better predict healing outcomes. To advance clinical decision-making, future research should focus on developing standardized, imaging-integrated classification systems with high interobserver reliability and proven prognostic value. Such systems would not only streamline treatment strategies but also reduce variability in patient outcomes, ultimately enhancing both conservative and surgical management of these common injuries.
